# *UGT1A1* Guided Cancer Therapy: Review of the Evidence and Considerations for Clinical Implementation

**DOI:** 10.3390/cancers13071566

**Published:** 2021-03-29

**Authors:** Ryan S. Nelson, Nathan D. Seligson, Sal Bottiglieri, Estrella Carballido, Alex Del Cueto, Iman Imanirad, Richard Levine, Alexander S. Parker, Sandra M. Swain, Emma M. Tillman, J. Kevin Hicks

**Affiliations:** 1 Department of Consultative Services, ARUP Laboratories, Salt Lake City, UT 84108, USA; ryan.nelson@aruplab.com; 2 Department of Individualized Cancer Management, Moffitt Cancer Center, Tampa, FL 33612, USA; alex.delcueto@moffitt.org; 3 Department of Pharmacotherapy and Translational Research, The University of Florida, Jacksonville, FL 32610, USA; nseligson@cop.ufl.edu; 4 Department of Hematology and Oncology, Nemours Children’s Specialty Care, Jacksonville, FL 32207, USA; 5 Department of Pharmacy, Moffitt Cancer Center, Tampa, FL 33612, USA; salvatore.bottiglieri@moffitt.org; 6 Department of Oncological Sciences, University of South Florida, Tampa, FL 33612, USA; estrella.carballido@moffitt.org (E.C.); iman.imanirad@moffitt.org (I.I.); richard.levine@moffitt.org (R.L.); 7 Department of Gastrointestinal Oncology, Moffitt Cancer Center, Tampa, FL 33612, USA; 8 Department of Satellite and Community Oncology, Moffitt Cancer Center, Tampa, FL 33612, USA; 9 College of Medicine, University of Florida, Jacksonville, FL 32209, USA; alexander.parker@jax.ufl.edu; 10 Georgetown University Medical Center, MedStar Health, Washington, DC 20007, USA; sandra.swain@georgetown.edu; 11 Indiana University School of Medicine, Indianapolis, IN 46202, USA; emtillma@iu.edu

**Keywords:** *UGT1A1*, pharmacogenetics, irinotecan, pazopanib, nilotinib, belinostat, cancer, genotype, precision medicine, Gilbert’s syndrome

## Abstract

**Simple Summary:**

The use of multi-gene testing platforms to individualize treatment is rapidly expanding into routine oncology practice. *UGT1A1*, which encodes for the uridine diphosphate glucuronosyltransferase (UGT) 1A1 enzyme, is commonly included on multi-gene molecular testing assays. *UGT1A1* polymorphisms may influence drug-induced toxicities of numerous medications used in oncology. However, guidance for incorporating *UGT1A1* results into therapeutic decision-making is sparse and can differ depending on the referenced resource. We summarize the literature describing associations between *UGT1A1* polymorphisms and toxicity risk with irinotecan, belinostat, pazopanib, and nilotinib. Resources that provide recommendations for *UGT1A1*-guided drug prescribing are reviewed, and considerations for implementation into patient care are provided.

**Abstract:**

Multi-gene assays often include *UGT1A1* and, in certain instances, may report associated toxicity risks for irinotecan, belinostat, pazopanib, and nilotinib. However, guidance for incorporating *UGT1A1* results into therapeutic decision-making is mostly lacking for these anticancer drugs. We summarized meta-analyses, genome-wide association studies, clinical trials, drug labels, and guidelines relating to the impact of *UGT1A1* polymorphisms on irinotecan, belinostat, pazopanib, or nilotinib toxicities. For irinotecan, UGT1A1*28 was significantly associated with neutropenia and diarrhea, particularly with doses ≥ 180 mg/m^2^, supporting the use of *UGT1A1* to guide irinotecan prescribing. The drug label for belinostat recommends a reduced starting dose of 750 mg/m^2^ for UGT1A1*28 homozygotes, though published studies supporting this recommendation are sparse. There was a correlation between *UGT1A1* polymorphisms and pazopanib-induced hepatotoxicity, though further studies are needed to elucidate the role of *UGT1A1*-guided pazopanib dose adjustments. Limited studies have investigated the association between *UGT1A1* polymorphisms and nilotinib-induced hepatotoxicity, with data currently insufficient for *UGT1A1*-guided nilotinib dose adjustments.

## 1. Introduction

Individualizing anticancer therapy based on genetic biomarkers is an essential component of precision oncology. There has been a rapid uptake of genetic testing to assist with the clinical management of cancer patients, due in part to strong evidence demonstrating associations between genetic polymorphisms and drug response. Inclusive are clinical data showing that certain germline polymorphisms can identify opportunities for targeted therapy, assist with mitigation of chemotherapy toxicity risks, and optimize supportive care pharmacotherapy [1,2,3,4,5]. Multi-gene pharmacogenetic panels or targeted next-generation sequencing platforms that provide somatic and germline information, rather than single-gene assays, are emerging as preferred genetic testing approaches in oncology. A limitation to multi-gene assays is that clinicians may be exposed to germline results where ambiguous recommendations exist for genotype-guided drug prescribing.

One such example is *UGT1A1*, which encodes for the uridine diphosphate glucuronosyltransferase (UGT) 1A1 enzyme. *UGT1A1* genetic variants can affect enzymatic function, causing reduced metabolic capacity. Dinucleotide repeats located in the gene’s promoter region are among the most frequently observed polymorphisms, with the UGT1A1*28 TA_7_ repeat occurring at a frequency of 0.09–0.41 in Asian populations, 0.26–0.32 in European populations, 0.37–0.4 in Latino populations, and 0.37–0.56 in African populations [6,7,8,9]. Dependent on ancestry, over 50% of individuals may harbor a *UGT1A1* polymorphism that can decrease enzymatic activity [6,7,8]. Table 1 provides example *UGT1A1* variants, their predicted impact on metabolic function, and phenotype frequencies among race and ethnic groups. A comprehensive overview of *UGT1A1* polymorphisms, allele frequencies, and predicted enzymatic function is provided by the Clinical Pharmacogenetics Implementation Consortium (CPIC), which publishes evidence-based, peer-reviewed guidelines for how to translate genetic test results into actionable prescribing decisions for affected drugs [6,10]. Individuals who are heterozygous for one decreased function allele (e.g., UGT1A1 *1/*28) are predicted to be intermediate metabolizers (IMs), and those who are carriers of two decreased function alleles (e.g., UGT1A1 *28/*28) are predicted to be poor metabolizers (PMs) (Table 1) [6]. For drugs that undergo UGT1A1-mediated glucuronidation as the major elimination pathway, such as irinotecan and belinostat, decreased UGT1A1 metabolic capacity caused by genetic variation may result in elevated drug concentrations that can increase the risk of drug-induced toxicities. 

In addition to drug metabolism, UGT1A1 also has a role in bilirubin elimination. Individuals who are UGT1A1 PMs (e.g., UGT1A1 *28/*28, UGT1A1 *6/*6) may display mild hyperbilirubinemia, referred to as Gilbert’s syndrome [13]. However, cases have been published demonstrating that some UGT1A1 PMs may be asymptomatic [13,14]. Individuals with Gilbert’s syndrome are estimated to have only 25–30% of normal UGT1A1 activity [15]. In rare instances, *UGT1A1* genetic variants can result in almost complete loss of UGT1A1 function leading to high levels of unconjugated bilirubin that cause severe and debilitating symptoms described as Crigler–Najjar syndrome [16,17]. Oncology agents, such as pazopanib and nilotinib, can also impair bilirubin elimination through inhibition of UGT1A1 function [18,19,20]. Prescribing drugs that inhibit UGT1A1 to patients carrying *UGT1A1* loss of function alleles may increase the risk of hyperbilirubinemia and liver toxicity [21,22]. 

**Figure 1:**
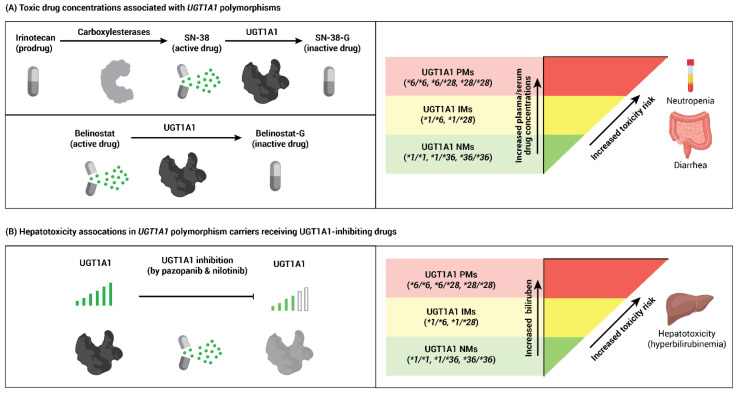
Association of *UGT1A1* polymorphisms with toxicity from cancer drugs. (**A**) Irinotecan and belinostat are metabolized by UGT1A1. Intermediate (IM) or poor (PM) UGT1A1 metabolic activity may result in greater than expected exposure to SN-38 (the active drug metabolite of irinotecan) and belinostat, increasing the risk of neutropenia or diarrhea. (**B**) The tyrosine kinase inhibitors pazopanib and nilotinib can inhibit UGT1A1 enzyme function, which may lead to an increased incidence of hyperbilirubinemia in UGT1A1 IMs or PMs.

Studies have investigated the association between *UGT1A1* polymorphisms and toxicity induced by irinotecan, belinostat, pazopanib, or nilotinib. UGT1A1 PMs, and potentially IMs, are proposed to be at an increased risk of diarrhea or hematologic toxicities due to elevated systemic exposure to irinotecan and belinostat (Figure 1A). Similarly, the inhibition of UGT1A1 by pazopanib or nilotinib has been reported to exacerbate hyperbilirubinemia in patients harboring *UGT1A1* genetic polymorphisms (Figure 1B) [20,23,24,25,26,27,28,29,30,31,32,33]. Findings from prior studies have led to FDA-approved labeling that provides specific irinotecan and belinostat dosing recommendations based on *UGT1A1* genetic test results and precautions for increased risk of pazopanib and nilotinib induced toxicities in those harboring *UGT1A1* polymorphisms [23,34,35,36]. However, clinical guidance for integrating *UGT1A1* results into cancer care are sparse and can be inconsistent. We reviewed the literature evaluating associations between *UGT1A1* polymorphisms and irinotecan, belinostat, pazopanib, or nilotinib toxicities along with applicability to patient care. Established resources for pharmacogenetic guidance were identified, and recommendations were evaluated for *UGT1A1*-guided therapy for irinotecan, belinostat, pazopanib, or nilotinib to elucidate further the role of *UGT1A1* in guiding cancer pharmacotherapy.

## 2. Methods

### 2.1. Study Design and Literature Review

A literature search was performed to identify studies analyzing the correlation between *UGT1A1* polymorphisms and irinotecan, belinostat, pazopanib, or nilotinib toxicity. Specifically, the PubMed^®^ database was searched from 1966 to June 2020 for the following keywords: (*UGT1A1* or *UGT1A* or Gilbert or uridine diphosphate glucuronidation) and (irinotecan or belinostat or pazopanib or nilotinib). Additional search terms for pazopanib included human leukocyte antigen (HLA) or *HLA-B* or HLA-B*57:01. Inclusion criteria for publications were meta-analyses, genome-wide association studies (GWAS), posthoc analyses, and clinical trials investigating the association between *UGT1A1* and clinical outcomes (e.g., diarrhea, neutropenia, hyperbilirubinemia, elevated alanine aminotransferase (E-ALT), dosage changes, or drug discontinuation). Studies included in this review were chosen considering the relevant characteristics from Thorn et al. [37]. 

Due to the large quantity of published data investigating the association of *UGT1A1* polymorphisms and irinotecan toxicity, many of which were retrospective studies consisting of small patient cohorts, we focused on meta-analyses and prospective studies that were not included in the meta-analyses identified investigating *UGT1A1*-guided irinotecan therapy. 

### 2.2. Pharmacogenetic Guidance Resources

There are several resources for genotype-guided pharmacotherapy recommendations, including the U.S. Food and Drug Administration (FDA), CPIC, National Comprehensive Cancer Network (NCCN), Dutch Pharmacogenetics Working Group (DPWG), European Medicines Agency (EMA), and peer-reviewed primary literature, including clinical trials and meta-analyses [10,38,39,40,41,42,43]. CPIC, DPWG, NCCN, FDA, and EMA were identified as established resources for information regarding genotype-guided cancer pharmacotherapy. Recommendations, or lack of recommendations, were collected for *UGT1A1*-guided irinotecan, nilotinib, or belinostat therapy, along with *UGT1A1*/HLA-B*57:01-guided therapy for pazopanib.

## 3. Results

### 3.1. Drug Concentration-Based Toxicity in UGT1A1 Polymorphism Carriers

#### 3.1.1. *UGT1A1*-Irinotecan

Irinotecan is a topoisomerase I inhibitor used to treat numerous cancer types, including gastrointestinal cancers, commonly as part of combination therapy with fluoropyrimidines. Irinotecan is a prodrug metabolized by carboxylesterases to the active metabolite SN-38, which has approximately 100-fold greater activity than the prodrug [25,44]. SN-38 is eliminated from the body through UGT1A1 mediated glucuronidation to SN-38-glucuronide [44]. UGT1A1*6 and *28 alleles and their impact on the incidence of irinotecan toxicity (severe neutropenia and diarrhea) caused by elevated exposure to SN-38 have been the most extensively studied, with the majority of evidence focused on the UGT1A1*28 allele [41,45,46,47,48,49]. Most studies investigating the interaction between *UGT1A1* variants and irinotecan have focused on non-liposomal irinotecan formulations. The impact of *UGT1A1* polymorphisms on liposomal irinotecan has not been fully elucidated, though some data supports an initial dose reduction for UGT1A1*28 homozygotes. [50,51].

Ten meta-analyses investigating the association between *UGT1A1* polymorphisms (i.e., UGT1A1*6 and *28) and irinotecan-induced toxicities were identified (Table 2). Although some studies suggested both UGT1A1 IMs and PMs were at increased risk of toxicity irrespective of the irinotecan dosage [48,52,53], the majority of data supports a “gene–drug exposure” interaction in which toxicities among *UGT1A1* polymorphism carriers were associated with higher levels of irinotecan exposure [52]. The strongest correlations with severe neutropenia and diarrhea were found among UGT1A1*28 homozygotes with irinotecan doses ≥ 180 mg/m^2^, particularly with doses ≥ 250 mg/m^2^ [41,52,54]. Hoskins and colleagues proposed that irinotecan-induced toxicity among UGT1A1 PMs was not significantly different than UGT1A1 normal metabolizers (NMs) at doses of less than 150 mg/m^2^ [52]. Irinotecan dosages that may increase the risk of toxicity among UGT1A1 IMs (i.e., UGT1A1 *1/*6 or *1/*28) have not been fully established. Some meta-analyses reported that for irinotecan doses ≥ 125 mg/m^2^, UGT1A1 IMs have a significantly higher risk for severe toxicity than UGT1A1 NMs [48,53,55,56]. However, other studies have not found statistically significant findings at doses ≤ 200 mg/m^2^ [48,54,57]. Evidence from the meta-analyses we identified suggests that UGT1A1 IMs may have a significantly higher risk of irinotecan toxicity than UGT1A1 NMs for doses ≥ 250 mg/m^2^. 

Recent prospective trials have investigated *UGT1A1*-guided irinotecan dosing (Table 3). Fujii et al. assessed the impact of prospectively reducing irinotecan doses by 20% for UGT1A1 PMs treated for colorectal cancer [61]. There were no differences in toxicities, disease response rate, or disease control rate for the patients who received a reduced irinotecan dose compared to UGT1A1 IMs or NMs. In the neoadjuvant setting, Catenacci and colleagues investigated preemptive dose reductions for irinotecan (180 mg/m^2^, 135 mg/m^2^, and 90 mg/m^2^ for UGT1A1 NMs, IMs, and PMs, respectively) as part of a FOLFIRINOX regimen. Margin-negative resection rates and pathological response grades did not differ among *UGT1A1* genotype groups. The authors also proposed that *UGT1A1*-guided therapy improved overall tolerability and cumulative dosing based on higher treatment completion rates than historical controls [61]. A phase I dose-finding study explored maximum tolerated doses of irinotecan in bevacizumab-FOLFIRI combination therapy [62,63]. UGT1A1 NMs tolerated a maximum irinotecan dose of 310 mg/m^2,^ whereas UGT1A1 IMs tolerated a maximum dose of 260 mg/m^2^. Results of the phase I study suggested that *UGT1A1* genotyping could identify patients who may tolerate higher doses of irinotecan. Overall, for the trials we identified that assessed disease response rates among prospective *UGT1A1*-guided irinotecan dosing regimens, there were no differences in outcomes between *UGT1A1* genotype groups. Additional prospective, large randomized studies are needed to elucidate further the impact of *UGT1A1*-guided irinotecan dosing on clinical outcomes, including toxicities and disease response. 

#### 3.1.2. *UGT1A1*-Belinostat

Belinostat is a histone deacetylase inhibitor approved for the treatment of relapsed or refractory peripheral T-cell lymphoma. In vitro experiments have demonstrated that UGT1A1 is the most prominent enzyme involved in belinostat glucuronidation, though UGT1A3, UGT1B4, and UGT2B7 also have significant roles in belinostat metabolism [71,72]. The FDA-approved drug label for belinostat recommends a reduced starting dose of 750 mg/m^2^ for UGT1A1*28 homozygotes, though published data investigating the influence of *UGT1A1* polymorphisms on observed toxicities is limited with most studies predominately focused on pharmacokinetic modeling [34].

A phase I trial investigated the effects of *UGT1A1* polymorphisms on the pharmacokinetics and toxicities (fatigue, nausea, vomiting, lethargy, neutropenia, and thrombocytopenia) of 48-hour continuous infusion belinostat. Belinostat drug exposure was significantly higher, as measured by half-life and area under the curve, for patients carrying UGT1A1 *28 or *60 decreased function alleles who received doses greater than 400 mg/m^2^/24 h [73]. UGT1A1 IMs or PMs receiving larger belinostat doses also had increased incidences of higher-grade neutropenia and thrombocytopenia. A pharmacokinetic model of 48-h continuous infusion belinostat did not find an effect of *UGT1A1* genotype status on platelet reductions, though the authors hypothesized that the data sets used had insufficient observations to predict differences [74]. 

Another phase I trial investigated the maximum tolerated belinostat dose combined with cisplatin and etoposide in patients with advanced small-cell lung cancer. The investigators observed an association between decreased belinostat clearance and UGT1A1 *28 or *60 carriers [75]. Those harboring UGT1A1 *28 or *60 alleles also experienced higher grade thrombocytopenia and elevated QTc intervals when compared to patients without *UGT1A1* polymorphisms [75]. A followed-up pharmacokinetic modeling and simulation study using data from both the phase I study of 48-h continuous infusion belinostat and the phase I study of belinostat in combination with cisplatin/etoposide suggested that a dose adjustment of belinostat 400 mg/m^2^/24 h for UGT1A1 IMs and 600 mg/m^2^/24 h for UGT1A1 NMs would provide equivalent exposures and potentially reduce toxicities for UGT1A1 IMs [76]. These studies also argued that the belinostat drug label should include dosing recommendations for other *UGT1A1* decreased function alleles besides UGT1A1*28.

### 3.2. Hepatotoxicity from UGT1A1-Inhibiting Drugs in UGT1A1 Polymorphism Carriers

#### 3.2.1. *UGT1A1* and HLA-B*57:01-Pazopanib

Pazopanib is a second-generation tyrosine kinase inhibitor indicated for use in patients with advanced renal cell carcinoma or advanced soft tissue sarcoma that have received prior chemotherapy. Pazopanib impedes the metabolism of bilirubin through direct inhibition of UGT1A1, and when prescribed to those harboring *UGT1A1* genetic variants, the incidence of hyperbilirubinemia is proposed to be higher. A total of 5 studies were found that investigated the influence of *UGT1A1* polymorphisms on hyperbilirubinemia in patients treated with pazopanib (Table 4). Two GWAS, including a total of 1486 patients from numerous phase II/III trials implicated *UGT1A1* polymorphisms to be significantly associated with total serum bilirubin [30,31]. In a subpopulation of patients who were genotyped for *UGT1A1* polymorphisms in the phase III COMPARZ study, UGT1A1*28, *37 or *6 homozygotes or inferred compound heterozygotes had higher baseline bilirubin and were more likely to experience hyperbilirubinemia (OR 9.97, 95% CI 4.13–24.03, *p* = 7.7 × 10^−8^) [32]. Similarly, two retrospective analyses of phase II/III studies reported a significant association between UGT1A1*28 homozygotes and hyperbilirubinemia risk with pazopanib [32,33]. 

Limited studies have explored using *UGT1A1* to guide pazopanib dosage. Henriksen et al. investigated the clinical utility of *UGT1A1* genotyping to guide dose adjustments for metastatic renal cell carcinoma patients treated with pazopanib who developed liver toxicity [20]. Of 261 patients in this study, 34 developed liver toxicity after a median of 29 days starting pazopanib. Eighteen of the 34 patients (53%) were UGT1A1 IMs, and 7 patients (21%) were UGT1A1 PMs. The median length of pazopanib interruption was 75 days for UGT1A1 PMs, 22 days for IMs, and 28 days for NMs. Pazopanib was restarted at very low doses for UGT1A1 PMs (median dose of 167 mg) and IMs (median dose of 217 mg). Of interest, *UGT1A1* polymorphisms were associated with improved outcomes, with UGT1A1 IMs having the longest median progressive free survival of 34.2 months followed by 22.3 months for PMs. There were limitations to this study, including the lack of a detailed algorithm for *UGT1A1*-guided dose adjustments and only a small subset of patients were *UGT1A1* genotyped.

Pharmacogenetic studies have also investigated whether polymorphisms in other pharmacogenes impact pazopanib toxicity. The HLA-B*57:01 allele has emerged as potentially influencing pazopanib toxicity. Pazopanib is proposed to interact with the HLA-B*57:01 binding cleft, leading to T-cell activation and increased incidence of immune-mediated hepatotoxicity in HLA-B*57:01 carriers [77]. Pazopanib-HLA-B*57:01 immune-mediated hepatotoxicity was assessed in a discovery cohort of eight phase II/III trials (*n* = 1188), a second confirmatory cohort of 23 additional phase I–III trials (*n* = 1002), and a GWAS for time to elevated alanine aminotransferase (ALT) (Table 4, Tables S1 and S2) [77]. For the combined discovery and confirmatory cohorts, HLA-B*57:01 was significantly associated with elevated ALT (*p* ≤ 5.4 × 10^−4^). Overall, HLA-B*57:01 carriers had a 1.5- to 2.0-fold greater risk for elevated ALT ≥ 3 times the upper normal limit. The GWAS meta-analysis for time to ALT ≥ 3 times the upper limit did not reveal any significant variant associations. Additionally, patients with both elevated ALT and hyperbilirubinemia were analyzed for HLA-B*57:01 and *UGT1A1* variants. No patients carried both risk alleles. 

#### 3.2.2. *UGT1A1*-Nilotinib

Nilotinib is a second-generation tyrosine kinase inhibitor used to treat patients with *BCR-ABL* positive chronic myelogenous leukemia (CML) [23,78]. Similar to pazopanib, nilotinib is also a potent inhibitor of UGT1A1, impeding the elimination of bilirubin. Those with *UGT1A1* polymorphisms prescribed nilotinib are proposed to have an increased risk of hyperbilirubinemia [79]. Retrospective analysis of a phase I/II clinical trial of nilotinib in patients with *BCR-ABL* positive CML or acute lymphoblastic leukemia (ALL) found that UGT1A1*28 homozygotes had a significant risk of grade 3/4 hyperbilirubinemia [28]. A population pharmacokinetics study of 493 patients with CML receiving nilotinib investigated the impact of *UGT1A1* variants on toxicity [29]. For UGT1A1 NMs, IMs, and PMs, high-grade hyperbilirubinemia occurred at 6%, 12%, and 48%, respectively. Furthermore, UGT1A1 PMs were more likely to develop high-grade hyperbilirubinemia at lower serum concentrations of nilotinib. However, not all investigations have found an association between UGT1A1 PMs and hepatotoxicity in patients receiving nilotinib [80]. 

Case studies have also reported nilotinib toxicities among UGT1A1 PMs. Assessment of UGT1A1 *6,*27, and *28 alleles in 34 Japanese patients with CML receiving nilotinib found that UGT1A1 PMs (*6/*6, *6/*28, and *28/*28) had increased rates of hyperbilirubinemia and greater nilotinib dose reductions [81]. A retrospective case-series including eight Japanese patients with CML receiving nilotinib found three UGT1A1 PMs (two *6 homozygotes, one *6/*28 compound heterozygote) experienced high-grade adverse events. In comparison, only two of the five UGT1A1 NMs experienced high-grade toxicities [82]. Single patient case reports have described similar findings of severe nilotinib-induced hyperbilirubinemia among UGT1A1 PMs [83,84,85,86]. Some of the studies identified in our review proposed that *UGT1A1* results may help avoid treatment delays and adverse events, but there is a lack of implementation studies assessing *UGT1A1*-guided nilotinib prescribing. 

### 3.3. Comparison of Pharmacogenetic Resources and Guidelines

CPIC, DPWG, EMA, FDA, and NCCN were identified as established pharmacogenetic resources to guide the application of genetic information to patient care. These resources were reviewed to determine if recommendations are provided for *UGT1A1*-guided irinotecan, belinostat, or nilotinib therapy, along with *UGT1A1*/HLA-B*57:01-guided therapy for pazopanib (Table 5). The FDA, DPWG, and EMA all recommend irinotecan dose reductions for UGT1A1*28 homozygotes, though specific dose reductions vary by resource. The FDA also provides specific recommendations for liposomal irinotecan with an initial starting dose of 50 mg/m^2^ (~30% dose reduction) for UGT1A1*28 homozygotes [50]. DPWG states that irinotecan dose adjustments for UGT1A1*28 heterozygotes (i.e., UGT1A1 *1/*28) are not warranted, with the other pharmacogenetic resources not providing any specific recommendations for UGT1A1*28 heterozygotes. CPIC guidelines for adjusting irinotecan dose based on *UGT1A1* status are currently not available, but CPIC categorizes *UGT1A1*-irinotecan as “level A” where the preponderance of evidence is deemed sufficiently strong that genetic information should be used to individualize pharmacotherapy [87,88]. NCCN, however, states that guidelines for using *UGT1A1* to guide irinotecan dosing in clinical practice have not yet been established. None of the identified pharmacogenetic resources provided information regarding other *UGT1A1* alleles. 

The FDA-approved drug label for belinostat provides specific dosing recommendations for UGT1A1*28 homozygotes, a dose reduction to 750 mg/m^2^ [34]. The drug label does not provide any specific recommendations for UGT1A1*28 heterozygotes or other *UGT1A1* alleles. None of the other pharmacogenetic resources currently provide guidance for belinostat dose adjustments based on *UGT1A1* results. *CPIC* categorizes *UGT1A1*-belinostat as level B, where evidence, though not as strong, supports that genetic information could be used to guided drug prescribing. 

The FDA Table of Pharmacogenetic Associations indicates that *UGT1A1* status may potentially impact pazopanib or nilotinib safety [40]. Specifically, UGT1A1*28 homozygotes may have a higher risk for pazopanib or nilotinib-induced hyperbilirubinemia. The FDA Table of Pharmacogenetic Associations also indicates that HLA-B*57:01 carriers may have an elevated risk for pazopanib-induced hepatotoxicity. *UGT1A1*-nilotinib and *UGT1A1*/HLA-B*57:01-pazopanib are categorized by CPIC as level B/C, indicating that evidence is not clear for supporting genotype-guided prescribing. None of the identified pharmacogenetic resources currently provide recommendations for using *UGT1A1* to guide nilotinib or pazopanib prescribing or HLA-B*57:01 status to guide pazopanib prescribing. 

### 3.4. Other Anticancer Drugs with Potential UGT1A1 Considerations

Our review focused on anticancer drugs that either have *UGT1A1*-guided prescribing recommendations provided by an established pharmacogenetics resource or anticancer drugs listed in the FDA Table of Pharmacogenetic Associations stating that *UGT1A1* status can potentially impact drug safety. The association between *UGT1A1* and drug toxicity has been investigated for several other anticancer drugs, including tyrosine kinase inhibitors such as gefitinib, erlotinib, and imatinib [89,90]. To date, evidence for these other anticancer drugs has not been sufficiently strong to warrant considerations for *UGT1A1* prescribing actions. Of interest, sacituzumab govitecan-hziy was recently approved to treat metastatic triple-negative breast cancer patients who have received at least two prior therapies for metastatic disease [91,92]. Sacituzumab govitecan is a Trop-2 directed antibody conjugated with the topoisomerase inhibitor SN-38. Based on clinical data to date, up to 72% of patients receiving sacituzumab govitecan have experienced grade 3/4 adverse reactions, including neutropenia (43%) and diarrhea (9%) [92]. The FDA-approved drug label states that UGT1A1*28 homozygotes have an increased risk of neutropenia, but limited data have been published regarding the association between *UGT1A1* and sacituzumab govitecan toxicity [93]. Further analysis of clinical trial data, including the ASCENT trial, may provide additional insights on whether *UGT1A1* polymorphisms influence sacituzumab govitecan toxicity [91,94,95]. 

## 4. Discussion

Utilizing genetic information to guide therapeutic decision-making in the oncology setting is rapidly becoming part of routine care. The exponential growth of commercially available anticancer drugs that target specific genetic mutations along with molecularly focused clinical trials are expanding treatment options for cancer patients. Furthermore, cancer patients have a high prevalence of exposure to drugs influenced by pharmacogenetic variants, with certain gene–drug interactions associated with severe and potentially life-threatening adverse events [88,96]. A piecemeal approach of testing one gene for one drug no longer reflects the clinical reality that multiple genetic variants can impact both anticancer regimens and supportive care therapies. As such, multi-gene panel testing inclusive of targeted next-generation sequencing platforms are emerging as preferred approaches for genetic testing in oncology. In certain instances, sequencing platforms can interrogate hundreds of genes and thousands of variants representing both somatic and germline findings [97,98]. In the not too distant future, whole-exome or whole-genome sequencing of the tumor and germline may become commonplace in oncology. A limitation of multi-gene assays is that clinicians may be exposed to vast amounts of genetic information that can potentially impact pharmacotherapy outcomes, but there may be a lack of guidance for applying to patient care for certain genes. We highlighted *UGT1A1* as an example focusing on irinotecan, belinostat, pazopanib, and nilotinib. 

Numerous studies have investigated the influence of *UGT1A1* on irinotecan toxicity, with evidence from meta-analyses supporting dose adjustments for UGT1A1 PMs receiving higher irinotecan doses to mitigate severe toxicities. The strongest correlations between *UGT1A1* and irinotecan toxicities have been observed with UGT1A1 PMs receiving doses ≥ 250 mg/m^2^, though doses these large are typically no longer used in clinical settings. The meta-analyses we identified in this review also support dose adjustments for UGT1A1 PMs receiving irinotecan doses ≥ 180 mg/m^2^. Although data support the clinical implementation of *UGT1A1* genotyping to guide dose adjustments for those receiving ≥ 180 mg/m^2^ irinotecan, the implications for lower irinotecan dosages have not been fully established. Prior studies have proposed that *UGT1A1* variants do not significantly influence irinotecan-induced toxicity for doses ≤ 150 mg/m^2^, with further analyses needed to determine the role of *UGT1A1*-guided therapy for lower irinotecan doses [52]. 

Irinotecan is commonly used in combination with other anticancer drugs that have similar adverse effects, which can potentially influence toxicity risks. In addition to UGT1A1 mediating elimination of the active metabolite SN-38, the parent drug irinotecan is metabolized by CYP3A4 [99]. Co-administration of drugs that strongly inhibit CYP3A4 or UGT1A1 can also increase exposure to SN-38 [35]. Thus, both gene–drug and drug–drug interactions can influence irinotecan toxicity risks. 

Yang et al.’s meta-analysis [59] was further analyzed by Hulshof et al. for validity and utility of pre-therapeutic genotyping of *UGT1A1* in Asian and Caucasian carriers of *6 and *28 alleles treated with irinotecan [99]. For *28 carriers, the number of patients that would need to receive dose reductions (number needed to treat) to prevent ≥ grade III neutropenia was 9, and to prevent ≥ grade III diarrhea was 14. The number of patients needed to be genotyped to prevent ≥ grade III neutropenia and ≥ grade III diarrhea was 79 and 127, respectively [100]. For *6 allele carriers, the number of patients that would need to receive dose reductions to prevent ≥ grade III neutropenia was 8, and to prevent ≥ grade III diarrhea was 11. The number of patients that would need to be genotyped to prevent ≥ grade III neutropenia and ≥ grade III diarrhea was 376 and 564, respectively [101]. 

In addition to utility, the value of preemptive *UGT1A1* testing has been reported as cost-effective and, in some instances, cost-saving [101,102,103,104,105,106]. The majority of data for cost savings, though, are from simulations rather than measuring actual healthcare costs in a prospective setting. Cost evaluations have traditionally focused on single gene–drug models, which are not reflective of current clinical realities that cancer patients are exposed to numerous drugs influenced by genetic variants [88]. Studies are emerging that multi-gene panels may have greater cost-effectiveness due to reuse of genetic test results [107,108]. Further studies are needed that incorporate multi-gene testing strategies and reuse of genetic results into cost-effectiveness models.

The majority of studies assessing the influence of *UGT1A1* polymorphisms on irinotecan therapy have been retrospective and focused on toxicities, with few studies investigating prospective *UGT1A1*-guided irinotecan dosing and impact on disease outcomes. Clinical trials exploring preemptive *UGT1A1*-guided irinotecan therapy are emerging with initial results suggestive of no differences in disease response rates for those who received a reduced irinotecan dose based on *UGT1A1* genotype [61,64]. Catenacci et al. proposed that reduced irinotecan doses for UGT1A1 PMs may result in higher treatment completion rates which could potentially improve treatment outcomes [64]. In contrast, UGT1A1 NMs may be underdosed. A dose-finding study suggested that the recommended dose of 180 mg/m^2^ for irinotecan in the FOLFIRI regimen was lower than the dose that can be tolerated by UGT1A1 NMs [63]. A follow-up phase II randomized trial compared the FOLFIRI regimen to a high-dose irinotecan FOLFIRI regimen in colorectal cancer patients, where UGT1A1 NMsin the high-dose FOLFIRI cohort received 300 mg/m^2^ irinotecan [66]. The overall response rate was significantly greater in the high-dose FOLFIRI cohort, and no differences in severe toxicities were observed, though there was no difference in survival between cohorts. Taken together, lower irinotecan doses for UGT1A1 PMs and higher irinotecan doses for UGT1A1 NMs may have the potential to increase disease response rates. Additional prospective, randomized studies to further elucidate the impact of preemptive *UGT1A1*-guided irinotecan dosing on clinical outcomes, including disease response, may further support the routine use of *UGT1A1* to guide irinotecan dosing. 

The FDA-approved drug label for belinostat recommends a reduced starting dose of 750 mg/m^2^ for UGT1A1*28 homozygotes, though we found limited published data supporting this specific dose recommendation. For the published studies assessing the impact of *UGT1A1* on belinostat toxicities, evidence was supportive of UGT1A1 PMs having an increased risk of hyperbilirubinemia. Similarly, evidence was supportive of *UGT1A1* variants being predictive of pazopanib or nilotinib toxicity. How to mitigate toxicity risks based on *UGT1A1* information is uncertain, as there appears to be limited data available to extrapolate pazopanib or nilotinib dose reductions based on *UGT1A1* status. Furthermore, clinical studies have correlated increased pazopanib exposure and occurrence of adverse events with improved disease outcomes, suggesting that pazopanib plasma concentrations for efficacy and toxicity overlap [109,110]. Taken together, there is insufficient evidence to recommend preemptive pazopanib or nilotinib dose reductions based on *UGT1A1* status. The presence of *UGT1A1* variants could help identify patients who may need closer monitoring due to toxicity risks. For those who develop hepatotoxicity, the drug inserts for pazopanib and nilotinib provide guidance for dose modifications. 

Arbitrio and colleagues recently described the complex process of pharmacogenetic biomarker validation and translation to clinical practice [111]. Sample size, study endpoints, and reproducibility are important considerations for biomarker discovery and validation. A limited number of studies were identified that assessed the impact of *UGT1A1* variants on pazopanib or nilotinib-induced hyperbilirubinemia. Furthermore, the majority of data consisted of subpopulations identified from clinical trials that were not directly investigating the impact of *UGT1A1* on these drugs. Reproducing results in larger study cohorts specifically designed to assess the impact of *UGT1A1* on pazopanib or nilotinib-induced hyperbilirubinemia would further validate *UGT1A1* as a pharmacogenetic biomarker for these drugs. Biomarker clinical utility demonstrated by improved patient management is also an important consideration [111]. There is currently a dearth of strong clinical data demonstrating *UGT1A1*-guided pazopanib or nilotinib prescribing improves patient care. The need for further pharmacogenetic biomarker validation and clinical utility studies supports our conclusion that there is currently insufficient evidence to recommend *UGT1A1* genotyping to guide pazopanib or nilotinib prescribing. The evidence supporting *UGT1A1*-guided irinotecan or belinostat dosing has been deemed sufficiently strong for regulatory bodies such as the FDA to provide specific dosing recommendations [34,35]. However, there is a lack of recommendations for performing *UGT1A1* genotyping before prescribing irinotecan or belinostat. Arbitrio et al. identified genotyping recommendations as a key consideration for supporting the clinical implementation of pharmacogenetic biomarkers [111]. 

The established pharmacogenetic resources that we identified as part of this review were mostly concordant that evidence is sufficiently strong to consider using *UGT1A1* to guide irinotecan dosing. However, one identified resource suggested that using *UGT1A1* to guide irinotecan dosing in clinical practice has not yet been established. Prior studies have described a lack of consensus for pharmacogenetic guidance across resources, which may hinder the integration of pharmacogenetics into patient care [112,113]. However, there are examples of collaborative efforts among pharmacogenetic resources to establish consistencies for genotype interpretations and clinical recommendations [114]. No pharmacogenetic resources provided *UGT1A1*-guided recommendations for pazopanib or nilotinib, and besides the FDA, no pharmacogenetic resources provided *UGT1A1*-guided recommendations for belinostat. 

For the pharmacogenetic resources that did provide *UGT1A1*-guided recommendations, they were all specific to UGT1A1*28. Other *UGT1A1* variants are predicted to result in decreased function, including UGT1A1*6 and *37. The UGT1A1*6 allele is more commonly observed in Asian populations, and when implementing *UGT1A1* genotyping into diverse patient populations, the UGT1A1*6 allele is likely to be observed [6]. Published data support that the UGT1A1*6 allele is associated with an increased risk of irinotecan-induced toxicity [58], and pharmacokinetic modeling suggests that other *UGT1A1* decreased function alleles besides UGT1A1*28 influence belinostat exposure [76]. For patients homozygous for other *UGT1A1* decreased function alleles who are predicted to be PMs, it may be reasonable to extrapolate dose adjustments from pharmacogenetic resources. Further research is needed to support implementation into diverse patient populations, as differences in enzymatic function among alleles may influence drug exposure. Following established processes for pharmacogenetic biomarker discovery and validation for less commonly observed *UGT1A1* alleles may provide the additional evidence needed to support translation into clinical practice [111]. Other considerations for implementing *UGT1A1* into patient care include the need for annotation of discrete results, electronic health record decision support tools, and provider education tools [115,116].

## 5. Conclusions

Evidence supports the use of *UGT1A1* information to guide irinotecan dosing, particularly for patients receiving doses ≥ 180 mg/m^2^. The drug label for belinostat recommends a reduced starting dose of 750 mg/m^2^ for UGT1A1*28 homozygotes, though we found limited published data supporting this specific dose recommendation. Evidence suggested that *UGT1A1* variants are predictive of pazopanib or nilotinib toxicity. However, there is insufficient evidence to recommend preemptive pazopanib or nilotinib dose reductions based on *UGT1A1* status.

## Figures and Tables

**Table 1:** Example *UGT1A1* alleles, predicted phenotype function, and frequencies among racial/ethic groups.

**Example *UGT1A1* Alleles and Predicted Function**						
**Star Nomenclature**		**Variant Type**		**Allele Function ^α^**		
UGT1A1*36		(TA)_5_		Increased Function		
UGT1A1*1		(TA)_6_		Normal Function		
UGT1A1*6		(211G > A)		Decreased Function		
UGT1A1*28		(TA)_7_		Decreased Function		
UGT1A1*37		(TA)_8_		Decreased Function		
**Predicted UGT1A1 Phenotypes Based on Commonly Observed Diplotypes**						
**Predicted UGT1A1 Phenotype**		**Frequently Reported Diplotypes** **[Less Commonly Investigated Diplotypes] ^β^**				
Normal Metabolizer (NM)		*1/*1 [*1/*36, *36/*36]				
Intermediate Metabolizer (IM)		*1/*28, *1/*6 [*1/*37, *6/*36, *28/*36, *36/*37]				
Poor Metabolizer (PM)		*6/*6, *6/*28, *28/*28 [*6/*37, *28/*37, *37/*37]				
***UGT1A1* Allele Frequencies Among Race/Ethnic Groups ^µ^**						
***UGT1A1*** **Allele**	**African American/** **Afro-Caribbean**	**Central/** **South Asian**	**East Asian**	**European**	**Latino**	**Sub-Saharan African**
*1	3%	54%	71%	36%	21%	49%
*6	<1%	4%	15%	<1%	1%	0%
*28	37%	41%	15%	32%	40%	40%
*36	8%	0%	0%	0%	0%	7%
*37	6%	0%	0%	<1%	0%	4%

**α**: *UGT1A1* allele function per CPIC and prior investigations [6,11]. **β:** While allelic diversity continues to be recognized, reference laboratories may only test for certain polymorphisms such as *1, *6, and *28. **µ:** Table recreated from CPIC *UGT1A1* Frequency Table [6,12].

**Table 2:** Meta-analyses investigating the pharmacogenetic influence of *UGT1A1* with the use of irinotecan.

*UGT1A1* Genotype	Dose	Endpoint	Major Findings ^a^	Conclusions
*28/*28 vs. *1/*1	>125 mg/m^2^	Diarrhea	OR 3.69, CI 2.0–6.38 (*n*) = 494	*28 allele carriers were at increased risk of severe diarrhea at doses > 125 mg/m^2^ [53].
*28/*28 vs. *1/*1	≤125 mg/m^2^		OR 0.43, CI 0.11–1.74 (*n*) = 99	
*1/*28 vs. *1/*1	>125 mg/m^2^		OR 1.92, CI 1.31–2.82 (*n*) = 9 30	
*1/*28 vs. *1/*1	≤125 mg/m^2^		OR 1.27, CI 0.67–2.42 (*n*) = 335	
*28/*28 or *1/*28 vs. *1/*1	>125 mg/m^2^		OR 2.06, CI 1.51–2.80, (*n*) = 1405	
*28/*28 or *1/*28 vs. *1/*1	≤125 mg/m^2^		OR 1.06, CI 0.57–1.99 (*n*) = 355	
*28/*28 vs. *1/*1	>150 mg/m^2^	Diarrhea	OR 2.37, CI 1.39–4.04 (*n*) = 774	*28 carriers (either heterozygote or homozygote) were at increased risk of neutropenia regardless of irinotecan dose. *28 homozygotes were at higher risk of diarrhea with doses > 150 mg/m^2^ [48].
*28/*28 vs. *1/*1	≤150 mg/m^2^		OR 1.41, CI 0.79–2.51	
*1/*28 vs. *1/*1	>150 mg/m^2^		OR 1.39, CI 0.97–1.98	
*1/*28 vs. *1/*1	≤150 mg/m^2^		OR 1.02, CI 0.7–1.50	
*28/*28 vs. *1/*28 or *1/*1	>150 mg/m^2^		OR 2.04, CI 1.23–3.38, (*n*) = 1317	
	≤150 mg/m^2^		OR 1.41, CI 0.82–2.43, (*n*) = 663	
*28/*28 vs. *1/*1	>150 mg/m^2^	Neutropenia	OR 4.64, CI 2.88–7.17 (*n*) = 764	
*28/*28 vs. *1/*1	≤150 mg/m^2^		OR 6.37, CI 2.69–10.71 (*n*) = 331	
*1/*28 vs. *1/*1	>150 mg/m^2^		OR 1.85, CI 1.32–2.58 (*n*) = 1189	
*1/*28 vs. *1/*1	≤150 mg/m^2^		OR 2.01, CI 1.21–3.34 (*n*) = 630	
*28/*28 vs. *1/*28 or *1/*1	>150 mg/m^2^		OR 3.34, CI 2.21–5.05, (*n*) = 1311	
	≤150 mg/m^2^		OR 3.63, CI 2.02–6.53, (*n*) = 704	
*28/*28 vs. *1/*1	50–100 mg/m^2^	Diarrhea	OR 5.93, CI 1.46–24.0	*6 carriers and *28 homozygotes were at a higher risk of diarrhea but not neutropenia [54].
*1/*28 vs. *1/*1			OR 1.33, CI 0.60–2.91	
*6/*6 vs. *1/*1			OR 17.64, CI 2.58–120.66	
*1/*6 vs. *1/*1			OR 4.36, CI 1.74–10.91	
*28/*28 vs. *1/*1		Neutropenia	OR 1.25, CI 0.2–7.95	
*1/*28 vs. *1/*1			OR 1.50, CI 0.86–2.62	
*6/*6 vs. *1/*1			OR 2.16, CI 0.28–16.96	
*1/*6 vs. *1/*1			OR 2.09, CI 0.66–6.62	
*28/*28	60–200 mg/m^2^	Neutropenia	OR 1.67, CI 0.94–2.97 (*n*) = 658	*6 and *28 may predict irinotecan-induced neutropenia, although additional confirmation is required [55].
*6/*28	30–350 mg/m^2^		OR 2.55, CI 1.82–3.58 (*n*) = 886	
*6/*6	60–200 mg/m^2^		OR 1.72, CI 0.97–3.04 (*n*) = 652	
*6/*6 vs. *1/*6 or *1/*1	60–350 mg/m^2^	Neutropenia	OR 3.276, CI 1.887–5.688 (*n*) = 984	*6/*6 and *6/*28 diplotypes were associated with an increased risk of neutropenia [56].
*6/*6 or *1/*6 vs. *1/*1			OR 1.542, CI 1.180–2.041 (*n*) = 994	
28/*28 or *6/*6 or *6/*28 vs. *1/*6 or *1/*28 or *1/*1			OR 3.275, CI 2.152–4.983, (*n*) = 923	
*28/*28 vs. *1/*28 or *1/*1	<150 mg/m^2^	Neutropenia	OR 1.80, CI 0.37–8.84, (*n*) = 229	Increased toxicity risk in *28/*28 carriers than *1/*1 or *1/*28 carriers at doses of irinotecan > 180 mg/m^2^. Similar risk at 80–125 mg/m^2^ doses of irinotecan across *28/*28, *1/*1, and *1/*28 carriers [52].
	150–250 mg/m^2^		OR 3.22, CI 1.52–6.81, (*n*) = 513	
	>250 mg/m^2^		OR 27.8, CI 4.0–195, (*n*) = 81	
*28/*28 vs. *1/*28 or *1/*1	<150 mg/m^2^	Neutropenia	RR 2.43, CI 1.34–4.39, (*n*) = 300	*28 homozygotes had a higher risk of neutropenia at all dose ranges [57].
	150–250 mg/m^2^		RR 2.00, CI 1.62–2.47, (*n*) = 1481	
	≥250 mg/m^2^		RR 7.22, CI 3.10–16.78, (*n*) = 217	
*1/*28 vs. *1/*1	<150 mg/m^2^		RR 2.94, CI 1.36–6.35 (*n* = 270)	
	150–250 mg/m^2^		RR 1.29, CI 1.04–1.62 (*n* = 1288)	
	≥250 mg/m^2^		RR 2.65, CI 0.7–9.95 (*n* = 180)	
*6/*6 vs. *1/*1	30–375 mg/m^2^	Neutropenia	OR 4.44, CI 2.42–8.14, (*n*) = 833	*6 carriage was associated with severe neutropenia, but only *6 homozygotes were at increased risk of diarrhea [58].
*1/*6 vs. *1/*1			OR 1.98, CI 1.45–2.71	
*6/*6 vs. *1/*1		Diarrhea	OR 3.51, CI 1.41–8.73	
*1/*6 vs. *1/*1			OR 1.44, CI 0.84–2.49	
*28/*28 vs. *1/*1	50–375 mg/m^2^	Neutropenia	OR 3.50, CI 2.23–5.50, (*n*) = 2609	*28 carriage was associated with increased risk of neutropenia and diarrhea, particularly for higher doses [59].
*1/*28 vs. *1/*1			OR 1.91, CI 1.45–2.50, (*n*) = 3516	
*28/*28 vs. *1/*1		Diarrhea	OR 1.69, CI 1.20–2.40, (*n*) = 1817	
*1/*28 vs. *1/*1			OR 1.45, CI 1.07–1.97, (*n*) = 2521	
*6/*6 vs. *1/*1	50–375 mg/m^2^	Neutropenia	OR 3.03, CI 2.05–4.47, (*n*) = 1466	*6 carriage was associated with increased risk of neutropenia and diarrhea, particularly for higher dosages [59].
*1/*6 vs. *1/*1			OR 1.95, CI 1.34–2.85, (*n*) = 1928	
*6/*6 vs. *1/*1		Diarrhea	OR 4.03, CI 1.98–8.32, (*n*) = 651	
*1/*6 vs. *1/*1			OR 1.98, CI 1.26–3.11, (*n*) = 844	
*28/*28 or. *1/*28 vs. *1/*1	50–375 mg/m^2^	Neutropenia	OR 2.15, CI = 1.71–2.70, *p* < 0.001 (*n*) = 5232	*28 allele carriers are at increased risk of severe diarrhea and neutropenia [60].
		Diarrhea	OR 2.18, CI = 1.68–2.83, *p* < 0.001 (*n*) = 4868	

^a^ Confidence intervals were 95% unless otherwise indicated. Abbreviations: CI, confidence interval; OR, odds ratio.

**Table 3:** Prospective studies investigating safety and efficacy of *UGT1A1* guided irinotecan dosing.

UGT1A1 Genotype	Dose	Major Findings ^a^	Conclusions
Group A: (*28/*28, *6/*6 or *28/*6) vs. Group B: (*1/*28 or *1/*6) vs. Group C: (*1/*1)	Initial dose: (group A: 120 mg/m^2^), (group B & C: 150 mg/m^2^) AVG adjusted doses: group A: (88.9 mg/m^2^) vs. group B: (99.7 mg/m^2^) vs. Group C: (105.4 mg/m^2^)	Incidence of thrombocytopenia for Group A was: 0 (0%) vs. Group B: 3 (14.3%) vs. Group C: 0 (0%), p = 0.045 (n) = 63	Initial 20% dose reduction for UGT1A1 PMs enhanced irinotecan safety and efficacy [61].
Group A: *1/*1, Group B: *1/28, Group C: *28/*28	group A: 180 mg/m^2^ (n = 19), group B: 135 mg/m^2^ (n = 16), group C: 90 mg/m^2^ (n = 1)	Margin-negative resection rates for groups A, B, and C were 89%, 94%, and 100%, respectively. Pathologic response grades 1, 2, and 3 were 36%, 25%, and 39%, respectively	UGT1A1-guided dosing was feasible with similar margin-negative resection rates and pathologic response grade across genotype groups [64].
Group A: (*1/*1) vs.Group B: (*1/*28) vs. Group C: (*28/*28)	Cohort 1: group A: 180 mg/m^2^ (n = 15), group B: 135 mg/m^2^ (n = 16), group C: 90 mg/m^2^ (n = 10)	DLTs: Group A: 2/15 (13%), Group B: 3/16 (19%), Group C: 4/10 (40%)	UGT1A1 guided dosing appeared to reduce toxicity in the *1/*28 group. [65].
	Cohort 2: Pancreatic (n = 19), and biliary tract cancer (n = 19) same dosing as cohort 1	DLTs: pancreatic cancer: 6/19 PTs (32%; 80% CI, 17.5–48.9%). Biliary tract cancer: 4/19 PTs (21%; 80% CI, 9.5%–37.8%)	
*1/*1 or *1/*28	HD: [300 mg/m^2^ for *1/*1 PTs (n = 13) and 260 mg/m^2^ for *1/*28 PTs (n = 27)], CG: [180 mg/m^2^ for *1/*1 PTs (n = 24), and 180 mg/m^2^ for *1/*28 PTs (n = 15)]	ORR for HD vs. CG: (67.5 vs. 43.6%; p = 0.001 OR: 1.73 [CI:1.03–2.93]). Severe toxicity incidence for HD vs. CG: (22.5% vs. 20.5%), dose reduction (22.5% vs. 28.2%), or prophylactic G-CSF (17.5% vs. 12.8%)	UGT1A1 genotyping may identify those who can tolerate higher doses of irinotecan for a more favorable ORR [66].
Group A: (*1/*1) vs.Group B: (*1/*28) vs. Group C: (*1/*6)	Initial dose: (groups A & B: 180 mg/m^2^), (group C: 120 mg/m^2^). AE < G3 AD 1: (groups A & B 210 mg/m^2^) vs. (group C: (150/m^2^) AE < G3 AD 2: (groups A & B : 240 mg/m^2^), (group C: 240 mg/m^2^). AE < G3 AD 3: (group A: 260 mg/m^2^)	>grade 3 neutropenia, fatigue, or diarrhea.	Trial completion is planned for October 2021 [67]
Group A: (*1/*1) vs.Group B: (*1/*28) vs. Group C: (*1/*6)	Initial dose: 165 mg/m^2^ with unspecified dose modification criteria (n = 30, 15, and 24 for groups A, B, and C, respectively)	Grade 4 neutropenia: group A: 4/30 (13%), group B and C: 18/39 (46%) (p = 0.0044). Neutropenia group A: (3/30: 10%) vs. group C: (8/24: 33%) (p = 0.0459). Dose modification requirement group A: 9/30 (30%), group B and C: 21/39 (54%) (p = 0.0549).	UGT1A1 polymorphisms were associated with neutropenia and febrile neutropenia. More dose modifications were required for heterozygous *6 and *28 carriers than wild-type carriers [68,69].
Group A: (*1/*1) vs. Group B: (*1/*28 or *28/*28)	125–180 mg/m^2^	PFS: [group A: 9.8 (CI: 8.6–10.9)] vs. [group B: (7.5 (CI:5.5–9.6) HR: 1.803 (CI: 1.217–2.671) p = 0.003] mOS [group A: 20.8 (CI: 18.7–23.0)], [group B: 13.3 (CI: 10.3–16.2) HR: 1.979 (CI: 1.267–3.091) p = 0.003], diarrhea: [group B vs. group A (OR: 2.673; CI 1.039–6.876)], neutropenia : [group B vs. group A (OR: 1.240; CI 0.554–2.776)]	UGT1A polymorphisms were predictive of survival outcomes and severe diarrhea in irinotecan-treated mCRC patients [70].
*1/*1 (n) = 25, and *1/*28 (n) = 23	260–370 mg/m^2^	mPFS: [9.0 (CI: 6.6–13.1 months)] ORR [33% (13 of 40 PTs)] DLT diarrhea: (5 of 13; 38%) DLT neutropenia: (6 of 13; 46%) MTD for *1/*28 PTs: 260 mg/m^2^ MTD for *1/*1 PTs: 310 mg/m^2^	MTD of genotype-directed irinotecan was 260 mg/m^2^ for *1/*28 PTs, and 310 mg/m^2^ for *1/*1 PTs. The most common DLTs were diarrhea and neutropenia [62]. Prior phase 1 study from same group reported MTDs of 370 mg/m^2^ and 310 mg/m^2^ in *1/*1 and *1/*28 PTs, respectively [63].

^a^ Confidence intervals were 95% unless otherwise indicated. Abbreviations: AE < G3 AD, adverse events greater than grade 3 adjusted dose; AVG, average; CG, control group; CI, confidence interval; DLTs, Drug limiting toxicities; G-CSF, granulocyte colony-stimulating factor; HD, high dose group; mCRC, metastatic colorectal cancer; OR, odds ratio, mPFS, median progression-free survival; ORR, overall response rate; OS, overall survival; PTs, patients; PFS, progression-free survival; PRG1, pathologic response grade 1; R0, margin-negative resection rate.

**Table 4:** Pharmacogenetic influence of *UGT1A1* or HLA-B*57:01 on hepatotoxicity with use of pazopanib.

Study Description	Major Findings ^a^	Conclusions
GWAS: Investigating pazopanib use in mRCC PTs (*n*) = 1099	*UGT1A1* polymorphisms were associated with total serum bilirubin (*p* = 2.9 × 10^−17^).	*UGT1A1* variants are associated with bilirubin elevation in pazopanib- treated PTs [30]
GWAS: Investigating pazopanib use in ovarian cancer PTs (*n*) = 387	*UGT1A1* polymorphisms were associated with serum total bilirubin (*p* = 1.1 × 10^−21^).	*UGT1A1* polymorphisms are associated with bilirubin elevation in pazopanib- treated PTs [31]
Clinical case-control study: Investigating pazopanib use in mRCC PTs (*n*) = 236	Of 38 PTs with hyperbilirubinemia, 32 (84%) were either UGT1A1*28/*28 (*n* = 18) or *1/*28 (*n* = 14). OR (95% CI) for developing hyperbilirubinemia was 13.1 (5.3–32.2) for *28/*28 PTs vs. other genotypes.	UGT1A1*28/*28 PTs receiving pazopanib are at greater risk of hyperbilirubinemia than *1/*1 and *1/*28 PTs [33]
Clinical case-control study: Retrospective analysis of phase III COMPARZ trial of mRCC PTs on pazopanib or sunitinib (*n*) = 369.	The incidence of hyperbilirubinemia was 17% (62 of 369) for PTs on pazopanib. UGT1A1 PMs were more likely to experience hyperbilirubinemia on pazopanib (*p* = 7.7 × 10^−8^) OR (95% CI) 9.97 (4.13–24.03)	UGT1A1 PMs prescribed pazopanib are at greater risk of hyperbilirubinemia than UGT1A1 NMs [32]
Retrospective, longitudinal cohort study of prospectively collected data: *UGT1A1*-guided pazopanib dose adjustments in mRCC PTs (*n*) = 261.	mPFS for *1/*1 PTs was 5.5 months (95% CI, 5.3–5.7) vs. *1/*28 and *28/*28 PTs 34.2 months (95% CI, 6.8–61.6) and 22.3 months (95% CI, not estimable), respectively. OS for *1/*28 and *28/*28 PTs was 16.6 months vs. 8.1 months for *1/*1 or unknown *UGT1A1*- status PTs (*p* = 0.03).	*UGT1A1* polymorphisms were associated with improved outcomes, despite pazopanib interruption and substantial dose reductions. [20]
GWAS and clinical case-control study: Meta-analysis of 31 clinical studies of pazopanib therapy. *HLA* genotyping + GWAS compared to transaminase levels. (*n*) = 1,188 in 1st cohort, (*n*) = 1002 in 2nd cohort.	In combined cohort (*n*) = 2190, HLA-B*57:01 carriage was associated with ALT elevation (*p* = 4.3 × 10^−5^ for MaxALT, *p* = 5.1 × 10^−6^ for time to ALT > 3× ULN event, *p* = 5.8 × 10^−6^ for time to ALT > 5× ULN event).	HLA-B*57:01 carriage confers a higher risk of ALT elevation in PTs receiving pazopanib [77]

^a^ Confidence intervals were 95% unless otherwise indicated. Abbreviations: CI, confidence interval; mRCC, metastatic renal cell carcinoma; OR, odds ratio, ORR, overall response rate; OS, overall survival; PTs, patients; PFS, progression-free survival; PGx, pharmacogenetic.

**Table 5:** Comparison of *UGT1A1* pharmacogenetic recommendations between guideline and administrative authorities.

Administrative Authority	Topic, Artifact, or Statement	Belinostat	Irinotecan	Nilotinib	Pazopanib
CPIC	CPIC level	B	A	B/C	B/C
	CPIC guideline	NR	NR	NR	NR
FDA	PGx associations with sufficient evidence to allow their use in guiding therapy management	May result in higher systemic concentrations and higher adverse reaction risk. Reduce starting dose to 750 mg/m^2^ for *28/*28 (PMs)	Results in higher systemic active metabolite concentrations and higher adverse reaction risk (severe neutropenia). Consider reducing the starting dosage by one level and modify the dosage based on individual patient tolerance for *28/*28 (PMs)	NR	NR
	Associations with data to suggest a potential impact on drug safety or response	NR	NR	Higher adverse reaction risk (hyperbilirubinemia) for UGT1A1 *28/*28 (PMs)	Higher adverse reaction risk (hyperbilirubinemia) for UGT1A1 *28/*28 (PMs)
DPWG	Recommendations	NR	UGT1A1 *28/*28: Start with 70% of the standard dose. If the patient tolerates this initial dose, the dose can be increased, guided by the neutrophil count.	NR	NR
NCCN	Recommendations	NR	Irinotecan should be used with caution in those with Gilbert’s disease. Guidelines for use in clinical practice have not been established.	NR	NR
EMA	Recommendations	NR	Recommends an initial dose reduction for UGT1A1 *28/*28 (PMs)	NR	NR

## Supplementary Materials

The following are available online at https://www.mdpi.com/article/10.3390/cancers13071566/s1, Table S1: Summary of findings from Xu et al.’s meta-analysis investigating the association between liver toxicity and HLA-B*57:01, Table S2: Summary of Xu et al’s meta-analysis of eight trials in the discovery, and 23 trials in the confirmatory, pharmacogenetic liver toxicity analyses with HLA-B*57:01.
