# Correction: Nelson et al. *UGT1A1* Guided Cancer Therapy: Review of the Evidence and Considerations for Clinical Implementation. *Cancers* 2021, *13*, 1566

**DOI:** 10.3390/cancers16213595

**Published:** 2024-10-25

**Authors:** Ryan S. Nelson, Nathan D. Seligson, Sal Bottiglieri, Estrella Carballido, Alex Del Cueto, Iman Imanirad, Richard Levine, Alexander S. Parker, Sandra M. Swain, Emma M. Tillman, J. Kevin Hicks

**Affiliations:** 1 Department of Consultative Services, ARUP Laboratories, Salt Lake City, UT 84108, USA; ryan.nelson@aruplab.com; 2 Department of Individualized Cancer Management, Moffitt Cancer Center, Tampa, FL 33612, USA; alex.delcueto@moffitt.org; 3 Department of Pharmacotherapy and Translational Research, The University of Florida, Jacksonville, FL 32610, USA; nseligson@cop.ufl.edu; 4 Department of Hematology and Oncology, Nemours Children’s Specialty Care, Jacksonville, FL 32207, USA; 5 Department of Pharmacy, Moffitt Cancer Center, Tampa, FL 33612, USA; salvatore.bottiglieri@moffitt.org; 6 Department of Oncological Sciences, University of South Florida, Tampa, FL 33612, USA; estrella.carballido@moffitt.org (E.C.); iman.imanirad@moffitt.org (I.I.); richard.levine@moffitt.org (R.L.); 7 Department of Gastrointestinal Oncology, Moffitt Cancer Center, Tampa, FL 33612, USA; 8 Department of Satellite and Community Oncology, Moffitt Cancer Center, Tampa, FL 33612, USA; 9 College of Medicine, University of Florida, Jacksonville, FL 32209, USA; alexander.parker@jax.ufl.edu; 10 Georgetown University Medical Center, MedStar Health, Washington, DC 20007, USA; sandra.swain@georgetown.edu; 11 Indiana University School of Medicine, Indianapolis, IN 46202, USA; emtillma@iu.edu

## Error in Table

In the original publication [1], there was a mistake in Table 1 as published. In certain instances, *UGT1A1* allele frequencies were inadvertently used to describe phenotype frequencies. The corrected Table 1 appears below. The authors apologize for any inconvenience caused and state that the scientific conclusions are unaffected. This correction was approved by the Academic Editor. The original publication has also been updated.

## Figures and Tables

**Table 1:** Example *UGT1A1* alleles, predicted phenotype function, and frequencies among racial/ethic groups.

**Example *UGT1A1* Alleles and Predicted Function**						
**Star Nomenclature**		**Variant Type**		**Allele Function ^α^**		
UGT1A1*36		(TA)_5_		Increased Function		
UGT1A1*1		(TA)_6_		Normal Function		
UGT1A1*6		(211G > A)		Decreased Function		
UGT1A1*28		(TA)_7_		Decreased Function		
UGT1A1*37		(TA)_8_		Decreased Function		
**Predicted UGT1A1 Phenotypes Based on Commonly Observed Diplotypes**						
**Predicted UGT1A1 Phenotype**		**Frequently Reported Diplotypes** **[Less Commonly Investigated Diplotypes] ^β^**				
Normal Metabolizer (NM)		*1/*1 [*1/*36, *36/*36]				
Intermediate Metabolizer (IM)		*1/*28, *1/*6 [*1/*37, *6/*36, *28/*36, *36/*37]				
Poor Metabolizer (PM)		*6/*6, *6/*28, *28/*28 [*6/*37, *28/*37, *37/*37]				
***UGT1A1* Allele Frequencies Among Race/Ethnic Groups ^µ^**						
***UGT1A1*** **Allele**	**African American/** **Afro-Caribbean**	**Central/** **South Asian**	**East Asian**	**European**	**Latino**	**Sub-Saharan African**
*1	3%	54%	71%	36%	21%	49%
*6	<1%	4%	15%	<1%	1%	0%
*28	37%	41%	15%	32%	40%	40%
*36	8%	0%	0%	0%	0%	7%
*37	6%	0%	0%	<1%	0%	4%

**α**: *UGT1A1* allele function per CPIC and prior investigations [6,11]. **β:** While allelic diversity continues to be recognized, reference laboratories may only test for certain polymorphisms such as *1, *6, and *28. **µ:** Table recreated from CPIC *UGT1A1* Frequency Table [6,12].
