# Supplementary material for: UGT1A1 Guided Cancer Therapy: Review of the Evidence and Considerations for Clinical Implementation
Source: Cancers (Basel). 2021 Mar 29;13(7):1566. doi: 10.3390/cancers13071566 (PMC8036652; doi:10.3390/cancers13071566)
Supplement: Supplementary file 1 [file cancers-13-01566-s001.pdf]

Supplementary Materials: UGT1A1 Guided Cancer Therapy: Review of the Evidence and Considerations for Clinical Implementation

Ryan S. Nelson, Nathan D. Seligson, Sal Bottiglieri, Estrella Carballido, Alex Del Cueto, Iman Imanirad, Richard Levine, Alexander S. Parker, Sandra M. Swain, Emma M. Tillmanand J. Kevin Hicks

Table 1. Summary of findings from Xu et al. 's meta-analysis investigating the association between liver toxicity and *HLA-B*57:01.*

Cohorts	Measurements	ALT >3 × ULN	Time to ALT >3 × ULN [b]	ALT >5 × ULN	Time to ALT >5 × ULN [b]	MaxALT
Discovery cohort	n (total) [a]	242	242	138	138	1,188
	*HLA-B**57:01 carriers: *n* (%)	23 (9.5)	23 (9.5)	17 (12.3)	17 (12.3)	64 (5.4%)
	*HLA-B**57:01 non-carriers: *n* (%)	219 (90.5)	219 (90.5)	121(87.7)	121 (87.7)	1,124 (94.6)
	Effect (95% CI)	OR = 2.3 (1.3–4.1)	HR = 2.4 (1.5–3.8)	OR = 3.1 (1.7–5.8)	HR = 3.6 (2.1–6.2)	1.6 (1.3–2.0)
	P	0.0039	4.8×10^{-4}	7.2×10^{-4}	4.1×10^{-5}	5.0×10^{-5}
Confirmatory cohort	n (total) [a]	187	187	93	93	1,002
	*HLA-B**57:01 carriers: *n*, (%)	18 (9.6)	18 (9.6)	7 (7.5)	7 (7.5)	67 (6.7%)
	*HLA-B**57:01 non-carriers: *n*, (%)	169 (90.4)	169 (90.4)	86 (92.5)	86 (92.5)	935 (93.3%)
	Effect (95% CI)	OR = 1.8 (0.93–3.5)	HR = 3.3 (1.7–6.2)	OR = 1.2 (0.45–3.1)	HR = 4.6 (1.7–12.6)	1.2 (0.97–1.51)
	P	0.086	8.1×10^{-4}	0.75	9.8×10^{-3}	0.085
Combined analysis	n (total) [a]	429	429	231	231	2,190
	*HLA-B**57:01 carriers: *n* (%)	41 (9.6)	41 (9.6)	24 (10.4)	24 (10.4)	131 (6%)
	*HLA-B**57:01 non-carriers: *n* (%)	388 (90.4)	388 (90.4)	207 (89.6)	207 (89.6)	2,059 (94%)
	Effect (95% CI)	OR = 2.0 (1.3–3.1)	HR = 2.5 (1.7–3.6)	OR = 2.1 (1.3–3.6)	HR = 3.4 (2.1–5.5)	1.4 (1.2–1.6)
	P	0.0014	5.1×10^{-6}	0.0058	5.8×10^{-6}	4.3×10^{-5}

a. Censored patients were patients that didn't experience an on-therapy ALT> ULN event; censored patients were used as strict controls. Patients who didn't experience an on-therapy ALT> ULN event and were not strict controls were excluded.

b. For time-to-event analysis, events were defined as the first ALT> 3 × ULN or >5 × ULN measured between the first day of pazopanib exposure through 28 days post-therapy.

Table 2. Summary of Xu et al.'s meta-analysis of eight trials in the discovery cohort and 23 trials in the confirmatory cohort, pharmacogenetic liver toxicity analyses with *HLA-B**57:01.

Discovery trials

Study ID	Clinical trial registration	Phase	Patient population	Pazopanib PGx, N
VEG102616	NCT00244764	II	LR/M RCC	129
VEG105192	NCT00334282	III	LA/M RCC	172
VEG107769	NCT00387764	III	LA/M RCC	50
VEG108844	NCT00720941	III	LA/M RCC	293
VEG113078	NCT01147822	III	LA/M RCC	76
VEG110727	NCT00753688	III	STS	89
VEG110655 (AGO-OVAR16)	NCT00866697	III	WOFPC	327
VEG114012	NCT01227928	III	WOFPC	52
**Total**				**1,188**

Confirmatory trials

Study ID	Clinical trial registration	Phase	Patient population	Combination agent for cancer	Pazopanib PGx, N
HYT109091	NCT00732420	I	ST	Topotecan	60
VEG10006	NCT00158782	I	ST	Lapatinib	38
VEG10007	NCT00401583	I	ST	—	23
VEG105424	NCT00387387	I	CRC	FOLFOX 6 or CapeOx	25
VEG105427	NCT00388076	I	BC	Paclitaxel or paclitaxel and carboplatin, or lapatinib and paclitaxel	40
VEG107200	NCT00370513	I	HC	—	13
VEG108925	NCT00540943	I	CRC	Cetuximab and irinotecan	11
VEG109599	NCT00678977	I	ST	Gemcitabine	20
VEG109603	NCT00722293	I	ST	Epirubicin or doxorubicin	71
VEG109607	NCT00619424	I	ST	Erlotinib or pemetrexed	40
VEG109693	NCT00722293	I	ST	Pazopanib alone or in combination with lapatinib	16
VEG102857	NCT00350727	I/II	RMG	Lapatinib	40
VEG104450	NCT00281632	II	OC	—	26
VEG105281	NCT00430781	II	CC	In combination with lapatinib or pazopanib monotherapy	104
VEG105290	NCT00367679	II	NSCLC	—	32
VEG108838	NCT00558103	II	IBC	Lapatinib	52
VEG109609	NCT00549328	II	NSCLC	—	13
VEG110190	NCT00561795	II	GT	Paclitaxel and carboplatin	12
VEG110264	NCT00849472	II	BC	Paclitaxel	80
VEG111109	NCT00866528	II	NSCLC	Paclitaxel	24
VEG111128	NCT00871403	II	NSCLC	Pemetrexed	59
VEG20007	NCT00347919	II	BC	Lapatinib	72
VEG113046	NCT01064310	III	RCC	—	131
**Total**					**1,002**

<u>**Abbreviations**</u>: **BC**, Breast cancer; **CapeOx**, capecitabine plus oxaliplatin; **CC**, Cervical cancer; **CRC**, Colorectal cancer; **FOLFOX**, folinic acid, fluorouracil, and oxaliplatin; **GT**, Gynecological tumors; **HC**, Hepatocellular cancer, **IBC**, Inflammatory breast cancer; **LA/M RCC**, Locally advanced or metastatic renal cell carcinoma; **LR/M RCC**, Locally recurrent or metastatic renal cell carcinoma; **NSCLC**, Non-small cell lung cancer; **OC**, Ovarian cancer; **PGx**, pharmacogenetics; **RCC**, Renal cell carcinoma; **RMG**, Relapsed malignant glioma; **ST**, Solid tumors; **STS**, Advanced soft tissue sarcoma progressed from prior treatment; **WOFPC**, Women with ovarian, fallopian tube, or primary peritoneal cancer whose disease had not progressed after completing standard debulking surgery and first-line chemotherapy.
